# Microbial Landscape of Pharmaceutical Failures: A 21-Year Review of FDA Enforcement Reports

**DOI:** 10.3390/biotech15010008

**Published:** 2026-01-18

**Authors:** Luis Jimenez

**Affiliations:** Biology and Horticulture Department, Bergen Community College, 400 Paramus Road, Paramus, NJ 07652, USA; ljimenez@bergen.edu

**Keywords:** microbial contamination, drugs, critical failure points, process control, aseptic processing, sterility, recalled drug

## Abstract

By analyzing Food and Drug Administration (FDA) enforcement reports from 2004 to 2025, we can determine the incidence of microbial contamination in non-sterile and sterile drugs in the United States of America and, at the same time, compare the trends and patterns over a period of 21 years to determine the distribution and frequency of microbial contaminants. The most common microorganisms detected from 2019 to 2025 were the mold *Aspergillus penicilloides*, with 17 citations for sterile products, followed by 16 citations for non-sterile products of *Burkholderia cepacia* complex (BCC) bacteria. Analysis from the last 21 years revealed the dominant microbial contaminants belong to the BCC, reaching a maximum level between 2012 and 2019. Some of the previous microbial contaminants, such as *Salmonella* and *Clostridium*, decline in the 2019–2025 period, with no notifications issued. *S. aureus* and *Pseudomonas* contamination persisted through the years but at very low levels. Gram-negative bacteria contaminated non-sterile drugs more frequently than Gram-positive. A worrisome trend continued with unacceptable levels of enforcement reports not providing any information on the identity of the microbial contaminant. New species of *Bacillus* and *Acetobacter nitrogenifigens* were responsible for a significant increase in non-sterile drug recalls. The main driver for sterile product recalls over a 21-year period is the lack of assurance of sterility (LAS) where major failures in process design, control, and operational execution were not conducive to the control of microbial proliferation and destruction. Enforcement data analysis identified the problematic trends and patterns regarding microbial contamination of drugs, providing important information to optimize process control and provide a framework for optimizing risk mitigation. Although the 21-year landscape demonstrated that some microbial contaminants have been successfully mitigated, others remain resilient. The emergence of new contaminants highlights the evolving nature of microbial risk. The consistent problem with LAS is not only a major regulatory violation but also a potential catalyst for the next major healthcare-associated outbreak.

## 1. Introduction

When pharmaceutical products are manufactured in the United States of America (USA) the federal government is responsible for the quality of the finished product formulation. The Food and Drug Administration (FDA) provides regulations and guidelines so the industry can produce safe, stable, and efficacious drugs [1]. Enforcing these regulations requires the release of warning letters and notices of violations to pharmaceutical companies when stability, safety, or efficacy have been compromised due to the lack of good manufacturing practices (GMPs). Of all the reasons for enforcement reports to be released, microbial contamination is a major problem because of the threat of pathogenic microorganisms to human health and the deterioration of the pharmaceutical formulation compromising stability and efficacy [2]. When products are contaminated by microorganisms in either hospitals, clinics, or in community environments because of failures during manufacturing, outbreaks leading to severe morbidity and mortality have been reported worldwide [3,4,5,6,7,8,9]. The ubiquitous presence of microbes in the environment requires validated containment systems in drug manufacturing facilities in both sterile and non-sterile products [1,2,10]. These systems must always be under constant monitoring and control due to the capacity of microorganisms to utilize a wide variety of inorganic and organic compounds to grow. Furthermore, most microorganisms in the environment are oligotrophic, e.g., thrive in low concentrations of organic carbon. Some microorganisms exhibiting that type of behavior are members of the *Pseudomonas* sp., *Acinetobacter* sp., *Burkholderia* sp., and *Aspergillus* sp., which exhibit a tremendous physiological versatility. Microbes’ tremendous genetic capabilities allow a rapid response to severe environmental fluctuations by developing survival strategies such as spore formation, viable non-culturable stages, and phenotypic and genotypic changes [2].

Drug manufacturing is a highly complex and specialized process. The presence, viability, and proliferation of microorganisms must be controlled during manufacturing. This control is based upon the optimization of air flow, pressure regulations, aseptic practices, temperature control, etc., where product manufacturing will take place [1,10,11,12]. When process control is lost, and systems fail to provide proper conditions to limit or eliminate microorganisms, objectionable microorganisms or high microbial counts in non-sterile products and the presence of microbial cells or products in sterile products can lead to serious health threats to consumers [2,13]. Underwood [14] clearly stated, “The microbiological quality of pharmaceutical products is influenced by the environment in which they are manufactured and by the materials used in their formulation”. When it comes to sterile drugs, the lack of terminal sterilization might lead to a microbial bioburden originating from the contamination of raw materials, equipment, air, personnel, and containers [15].

However, non-sterile pharmaceuticals are manufactured under conditions that minimize, rather than eliminate, microbial contamination, but the processes used during production are not monitored regularly [10,16,17]. The manufacturing of non-sterile pharmaceuticals is less stringent than that of sterile products, for example, in oral drugs or topical products (which are applied to body parts) where the formulations are not required to be sterile. However, federal regulations provide some information in the Code of Federal Regulations (CFR) part 211.113, highlighting the need for written procedures designed to prevent the presence of objectionable organisms from non-sterile drugs [16]. However, in aseptic manufacturing, there are specific regulations and guidelines to guarantee the sterility of the formulation and the constant monitoring of processes, water, air, and analysts [1,13,18]. The challenge of non-sterile manufacturing is that the products are not sterile, and systems are not as stringent as they are in sterile manufacturing [17]. Unfortunately, there are more chances for microbial excursions when systems are not under control [2,8,10,14].

Understanding the dynamics of microbial growth and the frequency of contamination in FDA enforcement reports will provide important information to optimize process control of pharmaceutical manufacturing. How frequent was microbial contamination? What were the genera and species of microorganisms detected and identified? What products were frequently contaminated due to the lack of process control during manufacturing? What were the trends and patterns of microbial contamination through the years? The first meta-analysis from published scientific studies, industry newsletters, and FDA enforcement reports revealed the predominance of bacteria belonging to the *Burkholderia cepacia* complex (BCC) in contaminated samples [2]. Mold and yeast contamination was also detected as the second-most common problem. However, mold and yeast identification was neglected with no genera or species revealed. Gram-negative bacteria were more abundant than Gram-positive bacteria in drug recalls. When it came to sterile drugs, the major reason for recalls was the lack of assurance of sterility (LAS). Other studies showed similar patterns when it came to microbial contaminants and reasons for recalls but concentrated on the analysis of the information exclusively obtained from FDA enforcement reports downloaded from the FDA website [17,19].

This study’s objectives are twofold. First, to ascertain the sources of microbial contamination in sterile and non-sterile pharmaceutical products from 2019 to 2025. Second, to compare these results with historical data back to 2004 to highlight any evolving risks and to determine any significant shifts in the dynamics of microbial contamination events leading to drug recalls [17,19]. Are there any new microorganisms causing contamination events? What are the trends and patterns over a 21-year period in FDA enforcement reports? The recall data was retrieved from the FDA website (https://www.accessdata.fda.gov/scripts/ires/index.cfm#tabNav_advancedSearch) (accessed on 1 December 2025) using the advanced search engine. The analysis focused specifically on sterile and non-sterile drug enforcement reports published from 1 July 2019, through 1 December 2025. After data collection, Microsoft Excel was used for data extraction and processing.

## 2. Bacterial Contamination of Non-Sterile Pharmaceutical Samples

When non-sterile drugs are manufactured, microbial enumeration testing must be performed [20,21]. Furthermore, the absence of objectionable microorganisms is also a requirement to prevent the health threat of highly pathogenic bacteria such as *Staphylococcus aureus*; *Salmonella* sp.; *Clostridium* sp.; *Escherichia coli*; yeast, e.g., *Candida albicans*; mold, e.g., *Aspergillus brasiliensis*; and the Enterobacteriaceae bacterial family [20]. These microorganisms are used during the validation of the microbial limits test (MLT) described in Chapters 61 and 62 of the United States Pharmacopeia (USP).

Examining the numbers of drugs recalled by the FDA from 2019 to 2025, among the USP microbial indicators, five recalls were due to the presence of different species of *Pseudomonas* and *S. aureus* (Table 1 and Table 2). Table 1 shows the historical data with recurrent microbial contaminants compared to the years 2019–2025, while Table 2 shows the frequency of new microbial contaminants described in enforcement reports from 2019 to 2025.

As shown in Table 1, three notifications were issued for contamination with *S. aureus*. All contamination events were found in a nasal wash system containing sodium bicarbonate and sodium chloride. Contamination with *S. aureus* has been consistently present throughout the entire 21-year span [17,19]. However, low levels of recalls were reported. *S. aureus* are Gram-positive cocci that are usually found in skin infections, respiratory infections, and gastrointestinal disorders. Some virulent strains of *S. aureus* known as methicillin-resistant *Staphylococcus aureus* (MRSA) are highly toxigenic and cause severe nosocomial infections [22].

Compared to previous reports (2012–2019) where drugs were recalled due to the presence of *Salmonella*, e.g., 28 recalls, there were no notifications issued for the presence of these extremely pathogenic bacteria [19]. Similar results were found with *Clostridium difficile*, where the numbers of recalls issued peaked during the same time period. Numbers decreased from 13 to 0 (Table 1).

In this study, the number of recalls issued for the presence of *Pseudomonas* species were limited to two (Table 1 and Table 2). One new microbial contaminant, e.g., *P. lactis*, was found in a saline nasal spray (Table 2), while a recurrent *Pseudomonas* sp. was detected in a tablet formulation (eletriptan hydrobromide) (Table 1). *Pseudomonas* numbers were higher for past years as previously reported [19]. The highest numbers, e.g., nine, were reported for the years 2004–2011 [17]. *Pseudomonas* bacteria are opportunistic pathogens mostly associated with respiratory infections and burns [23]. Other bacteria previously reported in high numbers, e.g., 45, were *Ralstonia pickettii*, but only one notification for a clobetasol propionate ointment formulation was issued in 2019–2025.

Bacteria belonging to the BCC, such as *B. lata, B. cepacia*, *B. multivorans*, and *B. contaminans,* showed the highest numbers of citations from 2019 to 2025. BCC bacteria were consistently isolated through the years, with the highest numbers in 2012–2019. However, when compared with previous enforcement data, there was a significant reduction in the recalls issued, with numbers decreasing from 105 (2012–2019) [19] to 16 (Table 1 and Table 2). There are several possible explanations for this reduction. Number one, it might have been that the constant reminder by the regulatory agencies of the threat that this group of bacteria poses to drug manufacturing, along with the widespread incidents reported around the world, led to more awareness by industry [10,24]. Number two, a new chapter in the USP, e.g., <60>, describes the tests for BCC bacteria, providing proper test methods [21,25]. Number three, industry is becoming more proactive regarding the continuous threat of BCC contamination when systems are not under control, possibly enhancing risk mitigation. BCC bacteria are ubiquitous in water, soils, and plants, and their persistence through the years indicates the possible health threat to immunocompromised patients and patients with health conditions such as cystic fibrosis and chronic granulomatous disease. However, serious cases of morbidity and mortality were reported in other patients when BCC bacteria were inadvertently introduced into the respiratory and circulatory system of patients by a contaminated drug [3,4,8].

What were the types of drug formulations contaminated by BCC bacteria in the current time period (2019–2025)? The drugs ranged from tablets (eletriptan hydrobromide), oral rinses (chlorhexidine gluconate), antimicrobial sanitizers (benzalkonium chloride (BZK), chloroxylenol), nasal spray (fluticasone propionate), gas relief (simethicone emulsions), external analgesics (camphor), topical anesthetics (lidocaine HCl), etc. Table 3 presents some of the reasons for recalls due to BCC contamination. What can we have inferred from the enforcement reports data presented in Table 3?

- Absence of or deficient cleaning procedures;
- Use of an unsuitable grade of water (use of potable water to clean equipment);
- Deficient water system controls;
- Deficient water system design;
- Absence of or deficient testing and specifications;
- Deficient manufacturing and packaging procedures;
- Absence of or deficient validation or lack of environmental monitoring in critical areas;
- Absence of qualified personnel with the proper education and training to perform and analyze microbiological data;
- Lack of process control.

Unfortunately, recent outbreaks caused by the contamination of BCC bacteria led to morbidity and mortality situations worldwide [6,7,26,27,28]. Although water, the most common raw material in pharmaceutical manufacturing, is a major source of contamination in most of the recalls, the lack of proper system validation and maintenance led to major microbial excursions. To prevent biofilm formation in water lines and equipment, rigorous chemical and thermal treatments must be validated to avoid system failure and product contamination [8,11,19,29]. The unusually large genome of some BCC bacteria, e.g., four chromosomes, provides the genetic capability to develop resistance to antibiotics, which is led by the secretion of beta-lactamases, the action of efflux pumps, and the low permeability of the outer membrane [30,31]. Enzymes such as monooxygenases (MOs) and dioxygenases (DOs) break down nitro aromatic and halogenated chemicals that are major active ingredients of many antipsychotic and analgesic drugs. Additionally, they can metabolize BZK, a common preservative and disinfectant, as a primary energy and carbon source [32].

Three new species of *Bacillus* were detected in non-sterile product recalls in the current time period. Two species, *Bacillus licheniformis* and *B. sonorensis*, showed equal numbers of citations, e.g., 10 (Table 2). However, *B. cereus* was reported in only five recalls. None of these *Bacillus species* were found in previous years. They were new contaminants. The formulations affected were burn creams, artificial tears ointments, wound care gels, nasal products, sucralfate, and atovaquone oral suspensions. There was a significant increase in spore-forming *Bacillus* species from 2 (2012–2019) to 26 recalls issued in the current time window. The primary health threat from drugs contaminated by *Bacillus* species is the probable opportunistic infections, especially in immunocompromised patients with preexisting conditions [33]. Of the three species detected in this time window, *B. cereus* is the most serious threat due to the production of toxins leading to severe diarrhea and possible systemic complications. The high incidence of this type of contamination might indicate the need to enhance the environmental monitoring protocol and the development of stricter controls to prevent moisture-related and airborne contaminants in the production facilities and processes [8,9,11].

Other notable new microbial contaminants in this time window were *Acetobacter nitrogenifigens*, with seven recalls, and *Chronobacter sakazakii* (four recalls) and *C. dublinensis* (one recall) (Table 2). All the recalls for *A. nitrogenifigens* were in laxative oral solutions of magnesium citrate saline. All the *Chronobacter* recalls were due to contamination in herbal powder which requires external application to the umbilical cord and skin of newborns for disinfection purposes. *Chronobacter* species can be opportunistic pathogens but can also become a serious health threat to infants and immunocompromised individuals [34]. They can be detected in the manufacturing environment and raw materials. On the other hand, *Acetobacter* species are not common human pathogens. However, three clinical cases were reported in patients with preexisting conditions [35].

## 3. Mold Contamination of Pharmaceutical Products

Compared to the years 2012–2019, there was a decrease in recalls by molds. The numbers went from 52 (2012–2019) to 33 (2019–2025) [19]. Higher citations were issued for non-sterile drugs, with 17, compared to 16 for sterile formulations. *Aspergillus penicillioides* was found to be the most common species reported, with 17 citations. Fungal contamination by *A. penicillioides* was found in 13 sterile and 4 non-sterile drugs. Most of the sterile products were based upon contamination of chlorhexidine gluconate and isopropyl alcohol solutions. Non-sterile drugs included sunscreens, antiseptics, cyclobenzaprine hydrochloride tablets, amifampridine tablets, nasal sprays, etc. Three other citations were issued due to the presence of the yeast *Candida parapsilosis* in a sodium fluoride oral rinse, the mold species *Penicillium brevicompactum* in a larotractenib oral formulation, and *Aspergillus sydowii* in a sunscreen formulation. Unfortunately, mold contamination led to severe systemic mycosis, sepsis, cornea infections, and endophthalmitis [5,36,37]. The investigation of a *A. penicillioides* outbreak in sterile products indicated that the storage conditions and breach in package integrity promoted fungal growth [37,38]. High heat and humidity allowed the multiplication of fungal cells with the potential health risk of systemic mycosis.

In some of the recalls, e.g., 13, the absence of identification of mold and yeast contamination was not conducive to a thorough root-cause analysis (RCA) [39,40,41]. However, there was an increase in the number of citations with identification with genus and species from 12% to 63% [19]. Previous studies also reported lower values of mold identification, 10% and 17%, in recalls showing neither genus nor species information [2,17]. Proper identification of fungal contaminants determines the source of the contamination, helps in the implementation of corrective actions to prevent future excursions, and optimizes risk assessment. In 2025, accurate mold identification can be performed using amplification and sequencing of internal transcribed spacer (ITS) regions and ribosomal genes [42]. When analyzing the FDA notifications from 2004 to 2025, the genus *Aspergillus* was found to be the major reason for contamination events, with 24 recalls. *Aspergillus* is a common mold found in soil, water, plants, pharmaceutical products, medical devices, and indoor environments [2,8,42]. Overall, mold and yeast contamination are a persistent problem throughout the 21-year period analyzed, along with poor identification practices.

## 4. Unidentified Microbial Contamination of Non-Sterile Pharmaceuticals

There is an identity crisis in non-sterile recalls. This is manifested by the numbers of enforcement reports without any microbial identification. No genus or species are named. Of 146 non-sterile recalls analyzed from 2019 to 2025, 76 (52%) did not have any microbial identification on the microorganisms responsible for the citations. Previous reports revealed a higher percentage of non-sterile recalls without identification, with 77% lacking any information about the genus or species of the microbial contaminant [19]. Sutton and Jimenez [17] reported 28% recalls without microbial identification for the time period of 2004–2011. The following were some of the reasons for recalls when identification was not provided:

- cGMP deviations: the product may have microbial contamination (10 citations).
- Microbial contamination of non-sterile product (39 citations).
- cGMP deviations: the firm reported possible microbial contamination in the purified water used in the manufacturing of the products. No contamination was found in the final products (four citations).
- cGMP deviations: microbial contamination was reported in stagnant water in the duct of the manufacturing equipment (13 citations).
- Microbial contamination: out of specification for microbial counts (four citations).
- Fungal or yeast contamination (eight citations).

Since non-sterile drug testing looks for bioburden levels and objectionable microorganisms, it was unclear if the recalls were due to high contamination levels or specific pathogens. Without accurate identification, assessing whether contamination is truly objectionable or poses a risk to consumers is extremely hard. A robust identification program will provide critical information to understand contamination sources and process deviations [39,40]. Furthermore, process remediation and optimization are based upon knowing the identity of the microbial contaminant. For instance, molds are known to be mostly related to air contamination or disinfection deficiencies, Gram-negative bacteria are related to water and raw materials, and Gram-positive bacteria can be related to personnel or air excursions [2,8,19]. How do you know that a microorganism is objectionable if you do not identify the genus and species? This lack of transparency hinders RCA.

Standard operating procedures (SOPs) and rigorous analyst training are the foundation of a functional microbial identification program. This should include basic microbiological procedures such as aseptic techniques; macroscopic features, e.g., looking at the plates with the naked eye to recognize whether the growth is bacterial or fungal; and microscopic analysis, e.g., Gram-staining, spore staining, and simple biochemical tests such as catalase testing, oxidase testing, and substrate utilization tests. Analysis of ribosomal 16S rRNA genes is the gold standard for identification of bacteria [9,43,44,45]. The 16S ribosomal gene is highly conserved and encodes the RNA component of the bacterial 30S subunit. By comparing unknown sequences against public databases, investigators can identify bacteria accurately down to the species level [46]. When discrimination between species such as BCC bacteria is a problem, other genes, such as *smpMB* and *recA*, are used to increase the resolution of the identification process [47]. From a medical perspective, an unidentified microorganism is a “blind threat”. If there is a patient with an infection caused by a recalled drug lacking microbial identification in the FDA enforcement report, it will restrict the doctor’s ability to carry out the following:

- Select appropriate antimicrobial therapy;
- Conduct accurate epidemiological tracking during the outbreak;
- Assess the long-term prognosis of the affected patient.

## 5. Microbial Contamination of Sterile Products and Lack of Assurance of Sterility

When the enforcement reports for sterile drugs were issued for non-sterility, 13 were because of contamination with *Aspergillus penicillioides*, as previously discussed. One recall was due to contamination with *B. cereus* in a lubricant eye ointment and another with *Paenibacillus lautus* in a sterile injection of human chorionic gonadotrophin. Other citations without microbial identification were as follows:

- Microbial contamination of sterile products: confirmed sterility failure identified during stability testing at the 12-month time point (one citation).
- Non-sterility: firm’s third-party lab confirmed microbial contamination (one citation).
- Elevated levels of endotoxin (four citations).
- CGMP deviations: environmental and personnel monitoring out-of-action limit excursions were not being properly investigated (33 citations).
- Non-sterility (41 citations).
- Non-sterility: the FDA analysis found unopened tubes to be contaminated with bacteria (three citations).
- Non-sterility: the FDA found insanitary conditions and positive bacterial test results from environmental sampling at the manufacturing facility (eight citations).

Non-sterility was the most cited violation, with 41 recalls, followed by out-of-limits excursions in environmental monitoring, with 33. The numbers of sterility failures were up from 95 (2012–2019) to 106 (2019–2025) [19]. This increase could have been due to an increase in regulatory oversight, pandemic-induced operations where manufacturers increased production capacity rapidly, and the increased complexity of aseptic manufacturing.

In the USA, testing of sterile drugs to ascertain the presence of microorganisms requires the use of two different enrichment media incubated at two different temperatures [15]. For aerobic microorganisms, the temperature is 25 °C, while anaerobic growth will be detected using 35 °C. The standard incubation time is 14 days. Positive microbial growth is indicated by an increase in the media turbidity. This traditional method is resource-intensive, requiring specialized laboratory facilities, stringent gowning protocols, and dedicated equipment. The primary indicator of microbial growth is turbidity; however, visual assessment is inherently subjective. Subtle growth indicators, such as a thin pellicle at the bottom of a container, can easily be overlooked without manual agitation. Furthermore, if the initial sample addition causes immediate turbidity in the medium, analysts cannot definitively confirm growth through visual means alone. In such cases, subculturing onto solid agar media is required, which significantly extends the testing timeline.

However, different technologies have been reported for rapid testing of sterile pharmaceutical products using ATP bioluminescence, solid phase laser scanning cytometry, and colorimetric sensors with reflected light to determine the amount of released carbon dioxide (CO_2_) [48,49,50]. Regardless of the methodology, completion of the RCA when microbial contamination is present will allow process optimization. The FDA strongly prefers the use of genotypic methods (DNA-based) over phenotypic methods (biochemically based) [1,9].

As per previous reports, enforcement data analysis identified a lack of assurance of sterility (LAS) as the primary driver for sterile drug recalls from 2019 to 2025 [2,17,19]. Because of the large database, the analysis was limited to 1000 enforcement reports. A diverse range of sterile injectables and therapeutic agents were recalled; these included, among others, basic saline and water for injection and more complex pharmaceutical formulations such as ophthalmic solutions, analgesics, antibiotics, and antineoplastic agents. The following were the reasons for recalls:

- Lack of assurance of sterility: bags have the potential to leak (three citations).
- Lack of assurance of sterility: product sterility cannot be guaranteed (32 citations).
- Lack of assurance of sterility: out-of-specification results for container closure test which cannot guarantee the sterility of the product (one citation).
- Lack of assurance of sterility: bags have potential leaks (three citations).
- Lack of assurance of sterility: products manufactured in a manner that cannot guarantee their sterility (23 citations).
- Lack of assurance of sterility: undefined GMP violations (641 citations).
- Lack of assurance of sterility: FDA inspection raised sterility assurance concerns (39)
- Lack of assurance of sterility: manufacturing areas for the recalled products exceeded acceptance levels for microbial recovery, leading to lack of sterility assurance for these sterile injectable products (15 citations).
- Lack of assurance of sterility: potential for a defective syringe cap, resulting in a non-integral unit (32 citations).
- Lack of assurance of sterility: due to concerns with production processes which cannot assure sterility of products intended to be sterile (22 citations).

Undefined GMP violations were the number one reason for recalls, as previously reported by Jimenez [19]. These undefined GMP violations might be the result of systemic failures discovered during the FDA inspections. What can we infer from the enforcement data when the reason is revealed? There seem to be three prevalent themes when it comes to LAS: poor personnel practices, complete loss of environmental and process control, and a very flawed operational design during manufacturing. Sterilization is a rigorous process designed to eliminate all microorganisms through physical and chemical methods [9,15,18]. However, because sterility testing is destructive, there is no way to test all units in a batch. Therefore, sterility is defined by the sterility assurance level (SAL). SAL is the benchmark for sterility and can be achieved by the validation of sterilization cycles and aseptic processing under strict GMP control of the manufacturing facility, processes, and personnel [18,51]. In pharmaceutical manufacturing, the standard for SAL is less than one non-sterile unit per million (10^−6^). For instance, for a product that begins with a bioburden of 10^3^ spores, a sterilization process with an inactivation factor of 10^−9^ is required to reach the 10^−6^ threshold. This guarantees that the likelihood of a microbial survivor is acceptably remote. Common sterilization technologies used to achieve these standards are as follows:

- Filtration;
- Steam sterilization (autoclaving);
- Dry heat sterilization;
- Ionizing irradiation;
- Ethylene oxide gas.

The choice of the sterilization method will be based on whether the product formulation and packaging can endure the process without compromising efficacy, safety, and stability [51]. Nevertheless, most of the drugs involved in the recalls were manufactured by aseptic processing. Aseptic processing is a more complicated manufacturing process when compared with terminal sterilization [1,13]. There are more chances of microbial contamination during the separation, purification, filling, and packaging of the drugs. Most sterile injectable drugs, such as biologics, advanced therapies, and small molecules, depend on aseptic manufacturing due to the fact that the chemical composition of the drug cannot tolerate terminal sterilization [52]. Enforcement reports revealed that the risk of microbial contamination exceeded acceptable limits, indicating fundamental failures in process design and control [1,13]. This is a recurrent problem over the years. Similar results were reported since 2007 [2]. Companies must address these systemic vulnerabilities by optimizing the following:

- Process design: improperly designed facilities and non-validated processes increased the risk of microbial survival. Deficient sterilization validation allowed the possibility of microbial survival and growth contaminating the finished drug product. An improperly designed environmental monitoring program increased the risk of introducing microorganisms into the process, resulting in major production losses.
- Operational execution: poor aseptic techniques compromised product safety, stability, and efficacy. Lack of validation of sterilization procedures endangers product quality and increases the risk of morbidity and mortality for patients.
- Quality oversight: deficient documentation to support process control and sterility.
- Physical integrity: questionable package integrity increased the probability of microorganisms compromising drug safety and potency.

## 6. Conclusions

Based upon information collected from FDA enforcement reports, the profile of microbial contamination in pharmaceutical manufacturing is evolving. While *Burkholderia cepacia* remains a persistent “legacy” threat in non-sterile drugs, the recent period (2019–2025) has seen a shift toward a wider variety of environmental molds (*Aspergillus penicilloides*) in sterile drugs and spore-forming bacteria in non-sterile drugs (*Bacillus* spp.). This shift suggests a need for updated environmental monitoring protocols and stricter controls against moisture-related and airborne contaminants in manufacturing facilities. Poor microbial identification practices continued to hinder risk mitigation and process control. Sterile drug recalls were mostly due to LAS, which is a recurrent problem throughout the 21 years analyzed, indicating poor understanding of basic process design, operational execution, quality oversight, and physical integrity. Optimization of non-sterile drug manufacturing will require the ability to control microbial bioburden to safe levels and avoid the presence of objectionable microorganisms that can pose a threat to consumers. Sterile drugs must be made free of any microorganisms or microbial products by relying on processes and systems that provide accurate and consistent levels of sterility assurance. FDA enforcement reports provided valuable information to understand the trends and patterns developed in a 21-year period to understand the microbial diversity in contaminated drugs in the USA and to prioritize practices to minimize emerging microbial risks.

## Figures and Tables

**Table 1:** Comparison of microbial contaminants in recalled drugs from 2004 to 2025.

Microorganism	2019–2025	Number of Recalls 2012–2019 [19]	2004–2011 [17]
*Achromobacter xylosoxidans*	0	1	1
*Aspergillus* spp.	0	4	1
*Aspergillus fumigatus*	0	1	0
*Bacillus circulans*	0	1	0
*Bacillus thuringiensis*	0	1	0
*Burkholderia cepacia.*	9	102	35
*Burkholderia contaminans*	2	1	0
*Burkholderia gladioli*	1	1	0
*Burkholderia multivorans*	1	1	0
*Clostridium difficile*	0	13	0
*Escherichia coli*	0	2	1
*Herbaspirillum huttiense*	0	1	0
*Klebsiella pneumoniae*	0	1	0
*Paecilomyces saturatus*	0	1	0
*Pseudomonas* spp.	1	1	2
*Pseudomonas aeruginosa*	0	2	7
*Ralstonia pickettii*	1	45	0
*Rhinocladiella similis*	0	1	0
*Salmonella*	0	28	2
*Sarcina lutea*	0	1	0
*Serratia liquefaciens.*	0	6	0
*Staphylococcus aureus*	3	2	1
*Staphylococcus saprophyticus*	0	4	0
*Staphylococcus warneri*	0	1	1
*Sphingomonas paucimobilis*	0	2	0
*Variovorax paradoxus*	0	1	0

**Table 2:** Identification of new microbial contaminants in recalled drugs from 2019 to 2025.

Microorganism	Number of Recalls
*Acetobacter nitrogenifigens*	7
*Aspergillus* *penicilloides*	17
*Aspergillus sidowii*	1
*Bacillus cereus*	6
*Bacillus licheniformis*	10
*Bacillus sonorensis*	10
*Burkholderia lata*	2
*Burkholderia* sp.	1
*Chronobacter sazaki*	4
*Chronobacter dublinensis*	1
*Enterobacter cloacae*	1
*Gluconacetobacter liquefaciens*	1
*Providencia rettgeri*	1
*Pluralibacter gergoviae*	1
*Paenibacillus lautus*	1
*Candida parapsilosis*	1
*Cohnella* spp.	1
*Penicillium brevicompactum*	1
*Pseudomonas lactis*	1
*Serratia marcescens*	1
*Terribacillus*	1

**Table 3:** Reasons for recalls of drugs due to BCC contamination.

Reason for Recall	Product Description
Microbial contamination of non-sterile drug product. The product was found to be contaminated with the bacteria *Burkholderia cepacia*.	Topical anesthetic hydrogel
Microbial contamination of non-sterile products: repackaged product was recalled by manufacturer because it was contaminated with the bacteria *Burkholderia lata*.	Chlorhexidine gluconate oral rinse
cGMP deviations; FDA inspection of manufacturing observed potential *Burkholderia cepacia* complex (BCC) contamination, inadequate cleaning, inadequate microbiological testing, and insanitary conditions.	Chlorhexidine gluconate oral rinse
cGMP deviations: test results confirmed that atypical *Burkholderia cepacia* were a result of personnel error.	Gas relief drops (simethicone/antigas)

## Data Availability

No new data were created or analyzed in this study.

## Abbreviations

The following abbreviations are used in this manuscript:

Abbreviation	Full Term
FDA	Food and Drug Administration
GMPs	Good manufacturing practices
CFR	Code of Federal Regulations
BCC	*Burkholderia cepacia* complex
MLT	Microbial limits test
USP	United States Pharmacopeia
RCA	Root-cause analysis
LAS	Lack of assurance of sterility
